# ‘Adoption’ by Maternal Siblings in Wild Chimpanzees

**DOI:** 10.1371/journal.pone.0103777

**Published:** 2014-08-01

**Authors:** Catherine Hobaiter, Anne Marijke Schel, Kevin Langergraber, Klaus Zuberbühler

**Affiliations:** 1 Centre for Social Learning and Cognitive Evolution and Scottish Primate Research Group, School of Psychology and Neuroscience, University of St Andrews, St Andrews, Scotland; 2 Department of Psychology, University of York, York, United Kingdom; 3 Institute Jean Nicod, Ecole Normale Supérieure, Pavillon Jardin, Paris, France; 4 Department of Anthropology, Boston University, Boston, Massachusetts, United States of America; 5 Department of Primatology, Max Planck Institute for Evolutionary Anthropology, Leipzig, Germany; 6 Department of Comparative Cognition, Institute of Biology, University of Neuchatel, Neuchâtel, Switzerland; 7 Budongo Conservation Field Station, Masindi, Uganda; CNRS (National Center for Scientific Research), France

## Abstract

The adoption of unrelated orphaned infants is something chimpanzees and humans have in common. Providing parental care has fitness implications for both the adopter and orphan, and cases of adoption have thus been cited as evidence for a shared origin of an altruistic behaviour. We provide new data on adoptions in the free-living Sonso chimpanzee community in Uganda, together with an analysis of published data from other long-term field sites. As a default pattern, we find that orphan chimpanzees do not become adopted by adult group members but wherever possible associate with each other, usually as maternal sibling pairs. This occurs even if both partners are still immature, with older individuals effectively becoming ‘child household heads’. Adoption of orphans by unrelated individuals does occur but usually only if no maternal siblings or other relatives are present and only after significant delays. In conclusion, following the loss of their mother, orphaned chimpanzees preferentially associate along pre-existing social bonds, which are typically strongest amongst maternal siblings.

## Introduction

Adoptions of orphaned infant and juvenile chimpanzees have been recorded at all long-term research sites [1]–[5]. In East African chimpanzee communities, adoption has been documented for older maternal siblings, nulliparous and infertile females [2]–[4] and by a maternal grandmother [5]. In contrast, in the West African communities of Taï Forest, Ivory Coast, adoptions by apparently unrelated group members are common, including adult males (one father) and parous females, particularly allies of the deceased mother with no known kin-relationship [1]. As in humans, adoption in chimpanzees involves the regular provision of allomaternal care, such as carrying, sharing food, defending, and grooming [1], [2], by an adult individual in ways that do not differ from what is normally provided by the biological mother [1]. These observations led Boesch et al. [1] to suggest that adoption by wild chimpanzees should be interpreted as a potential example of altruistic behaviour in the animal kingdom, mainly because of the significant ‘costs’ to the adopter [1], [5].

The understanding of prosocial behaviour in non-human animals, and in particular altruism, has been hampered by a failure to establish and implement clear behavioural definitions [6]–[8]. We take prosocial behaviour to be a ‘behaviour that increases the direct fitness of another individual’ [9]. Although recent research has provided within-species comparisons of prosocial behaviour, the emerging picture still remains unclear [see 10]. For instance, in chimpanzees there is evidence both for [11], [12] and against [13] prosocial behaviour. One explanation may be that the expression of prosocial behaviour is task and situation specific. For example, in contrast to wild chimpanzees, captive individuals may not actively share food, but do help others to complete a food reward task [14].

Altruism is one possible motivation for prosocial behaviour, although there are other possibilities. An accepted evolutionary way to define behaviour, such as adoption, as *altruistic* is in terms of its lifetime fitness consequences [7], [8]. For adoption to be an altruistic behaviour there must be an average ‘cost’ to the lifetime fitness of the adopter, and an average ‘benefit’ to the lifetime fitness of the orphan [8]. Boesch et al. [1] employ a definition for adoption that is based on immediate costs (to the adopter) and benefits (to the orphan) during the care period. However, as they note, adoption may also result in long-term benefits for the adopter, for example by gaining a future social ally [1]. If the initial cost to the adopter during the care period is met or exceeded by later benefit, adoption may be better described as mutualism [7].

Whether or not chimpanzees express prosocial, altruistically motivated behaviour has considerable implications for theories of human evolution, but unfortunately not many studies have addressed this question in free-ranging communities. A particular issue in the Boesch et al. study of adoption was that the researchers were unable to demonstrate that adoption clearly benefited the orphaned chimpanzees, as it did not increase their survival rate compared to non-adopted orphans [1]. In the Taï communities, as Boesch et al. discuss, this was likely due to the overall high mortality rates in the group over the two decades of the study [1].

Here, we revisit these questions with a study on adoption in a wild chimpanzee community with lower mortality rates, the Sonso chimpanzees of Budongo Forest, Uganda. In Budongo, unlike Taï, chimpanzees are not exposed to significant predation pressure (no confirmed sightings of large predators such as leopards since the onset of long-term research in 1992 and no direct human hunting of primates [15]), nor have there been any confirmed cases of death from anthropogenic disease [15]. Chimpanzee deaths as a result of human-wildlife conflict over crop-raiding have been recorded, as have deaths from snare-traps laid with the intention of catching other prey, such as antelope or bush-pigs, but in general, mortality rates in this community are relatively low. Because these low mortality rates impact on the number of orphans, and therefore on the number of opportunities for adoption available for analysis, we combined the new data from our own long-term records with those extracted from the published records of two other long-term East African chimpanzee research sites, Gombe and Mahale, in which no systematic analysis of adoption has taken place. For the Sonso community (and for other sites where data was available) we report all potential cases of adoption, including non-adopted orphans. We particularly consider the behaviour of kin, such as maternal siblings, versus non-kin towards orphan immature chimpanzees.

## Method

### Ethical Statement

This was a purely observational study that did not contain any interventions, and researchers had no interaction with the chimpanzees. All research adhered to the ethical ASAB/ABS Guidelines for the Use of Animals in Research and was conducted in compliance with the applicable national laws (Uganda Wildlife Authority and Ugandan National Council for Science and Technology; research permit reference: NS179).

### Study site and subjects

The Budongo Conservation Field Station (BCFS, formerly Budongo Forest Project, [15]) was established in 1990 in the Budongo Forest Reserve, situated in the western Rift Valley in Uganda (1°35′–1°55′N, 31°18′–31°42′E) at a mean altitude of 1050 m. The 793 km^2^ reserve includes 482 km^2^ of continuous, medium altitude, semi-deciduous forest [16] with an estimated population of around 600 chimpanzees [17] split into an estimated 6–10 communities. The Sonso chimpanzee community is located towards the centre of the reserve. Their territory contains one forest-edge boundary and shares boundaries with 3–4 other chimpanzee communities. Regular daily observation of the Sonso chimpanzee community started in 1991 and has been continuous until present. As of September 2012, the community included 69 individually recognised group members; 11 adult males, 24 adult females, 5 sub-adult males, 9 sub-adult females, and 20 juveniles and infants. Adults were defined as individuals above 15 years of age; sub-adults as between 10 and 15 years and regularly seen without their mothers (or adoptive carer); juveniles as 5 to 9 years of age, and infants as under 5 years of age [15]. For comparison with Boesch et al.’s study of adoption [1] we consider any immature individual (<12 years old) that is permanently associated with his/her mother to be *dependent*.

### Protocol and definitions

#### Maternal death and status as an orphan

Chimpanzee mothers are closely associated with their dependent offspring, while chimpanzee fathers do not typically associate with them [18], [19]. Thus, an individual chimpanzee is considered an *orphan* following the death of their mother, even if its father is present within the community. Under field conditions, even within a well-habituated community such as Sonso, it is not possibly to monitor all individual chimpanzees on a daily, or even monthly, basis. Therefore, where an individual’s death is not directly observed it may never be confirmed. Given the size of the chimpanzees’ home range and density of their habitat the carcass may either never be discovered, or may be discovered in a state of decomposition which prevents individual identification. Instead, death is usually inferred from unusually prolonged periods of absence from the community. In the Sonso community, we discriminated between ‘core’ and ‘peripheral’ females, based on how often they could be observed. Core females were regularly encountered, at least once per month, and they often travelled with the adult males. Peripheral females tend to forage in the outer parts of the community’s home range and can be absent (i.e. not seen by a researcher or field assistant), together with their dependent children, for several years. We employed a conservative estimate and considered a female as deceased if she had not been seen for >6 months (core females) or >4 years (peripheral females) or if her dependent infant and juvenile offspring returned to the community without her for a period of one-week or more.

All dependent offspring (<12 years old and permanently associated with his/her mother) are considered to be a candidate for adoption following the death (or assumed death) of his/her mother.

#### ‘Allomaternal care’ and ‘Adoption’

We define *allomaternal care* as the nurturing behaviour normally provided by the biological mother to her dependent offspring, such as carrying, sharing food, defending, or grooming. We employ Boesch et al.’s [1] definition of *adoption* as the provision of species-specific allomaternal care to an orphan by an adult or mature individual (>12 years old) for at least a two-month period. Again as per Boesch, for adoption to occur, we require that “…the adult be permanently associated with the orphan, as well as, at the very least, wait during travel for, provide protection in conflicts to, and share food with the orphan.” [1].

In addition to data from the Sonso and Taï forest chimpanzee communities we also reviewed data from the long-term chimpanzee research sites of Gombe and Mahale for which brief reports of adoption events have been published [2]–[5]. Furthermore, we reviewed the published long-term records of these communities in order to collect the demographic data on births, deaths and biological relationships [2], [3], [20].

No specific definition for adoption was provided for the Gombe data reviewed [2], [5]. One possible source of variation from the definition employed above is the use of the term adoption towards orphans who did not survive for >2-months beyond the death of their mother, thus allomaternal care was not always provided for a minimum of 2-months in these cases. In the Mahale data reviewed [3], [4], adopting individuals ‘transported, groomed, protected, and slept with the orphans’ and ‘provided all maternal care except lactation’; adoption of all three orphans described here lasted for over 2-months [3].

In addition to adoption following the mother’s death, Uhera & Nyundo [4] describe ‘temporary adoption’ in the Mahale community. Here a mother is separated from a dependent infant who is normally permanently associated with her. We consider ‘temporary adoption’ to be the provision of allomaternal care by an individual other than the mother for a period of 2 or more days, but less than two months. These cases do not require that the mother be dead.

As a further distinction, in order to provide a full description of chimpanzee behaviour in relation to orphans, we provide data on cases where following the death of the mother an immature individual (<12 years old) provides the allomaternal care required for adoption as defined by Boesch et al. [1]. As all of these cases involve siblings ‘adopting’ their younger siblings, we term this ‘immature sibling adoption’. Although siblings may provide some aspects of care to their younger siblings while their mother is alive, the mother is almost always the primary caregiver of all her dependent offspring while alive (but see Wroblewski et al. [5] for an exception). Following her death, ‘immature sibling adopters’ continue to permanently associate with their younger siblings and become their primary caregiver.

### Data collection

Since the onset of research at the Budongo Conservation Field Station in 1991 [15], field assistants have been recording party composition, ranging behaviour, and the frequency and duration of key social behaviours between individuals, such as grooming and aggression. Cases of adoption and associated behaviour in the Sonso chimpanzee community were extracted from long-term data sources. Researchers and field assistants keep a logbook for the purpose of collating unusual or rare observations; this record includes all deaths and adoptions between 1991 and 2013, and more detailed reports of allomaternal care provided to orphaned infants between October 2007 and January 2013. In addition, we interrogated the six highly experienced chimpanzee field assistants, two of whom have worked with the Sonso community for over 20 years.

### Genetic analyses

Long-term observational records (>20 years) were available for the four field sites in the cross-site analysis (Sonso, Taï, Gombe, Mahale [2], [3], [20]), allowing for the easy assignation of an individual as the mother or as a maternal sibling of an orphaned individual, and in some cases as a grandmother, or maternal aunt. In contrast, the determination of paternity (and therefore paternal relatives, such as siblings) through DNA is a relatively recent advance and data are often unavailable or patchy. We employ the term *non-kin* to refer to individuals that are neither the mother nor a maternal sibling of the orphan and that have no known paternal or maternal relationship to it.

We genotyped the Sonso chimpanzees at 7–19 autosomal microsatellite and, for males, also 13 Y-chromosome microsatellite loci, following procedures described in previous publications [21]–[23]. Briefly, we noninvasively collected chimpanzee faecal samples, which were first processed with a two-step ethanol-silica method, before extracting DNA using the QIAamp DNA stool kit with slight modifications of the manufacturer’s (QIAGEN) protocol [24]. We then used a two-step amplification method, where we initially combined all primer pairs with template DNA in a multiplex PCR followed by dilutions of the resultant PCR products for amplification of each individual locus, using fluorescently labelled forward primers and nested reverse primers in singleplex PCR reactions [25]. Paternity was assigned through likelihood-based methods implemented in the program CERVUS [26] and for male offspring, through Y-chromosome haplotype sharing. CERVUS analyses were conducted with the following parameters: 10,000 simulated offspring, 0.01 mistyping error rate, genotypes 0.95 complete, and offspring-specific values for the number of candidate sires and the proportion of candidate sires that were sampled. For offspring that were born in the community, candidate sire information was obtained from demographic records. For offspring that may have emigrated into the community (i.e. adolescent females), we set the number of candidate fathers as 100, and set the number of these sampled according to the number of Sonso candidate fathers that were present at the estimated birthdate of the offspring. All Sonso males estimated to be ≥8 years at the time of conception were considered to be candidate fathers. All paternity assignments achieved the 95% level of confidence based on LOD scores.

### Analysis of published data from other long-term field sites

Cases of orphaned individuals reported at other sites were examined for the following details: age of orphan, presence of maternal sibling in community, age of adopter (in years), any known kin-relationship of adopter to orphan, time before care was provided (in months), and survival of orphan after one year. Where data were unavailable these cases were excluded from the relevant analyses. In cases where the adoption or care was described simply as ‘fast’ or ‘quick’, we assigned an estimate of 1-week. In a small number of cases in the Taï communities (where mortality is high), single individuals adopted more than one orphan, although this never occurred simultaneously. In one case an individual could have contributed more than one data point to an analysis, in this case we calculated and used a single mean value. For further details of individual Sonso case histories see Supplementary Information in File S1 and Table S1.

## Results

During a 21-year observation period of the Sonso community (1991–2012), 18 females died or were presumed dead due to long-term disappearances. Seven had dependent immature offspring (<12 years) at the time of death, a total of N = 11 immature orphans (Table S1).

No adoption took place for 4 of the 11 orphans. Two of them simply disappeared while the other two, a maternal brother (10 years) and sister (4 years), were not adopted and did not receive care from any other individual, nor provided care to each other. The maternal sister managed to survive for less than 2 years following her mother’s death but then disappeared and presumably died. The maternal brother was already observed to associate and travel with the community males on a daily basis for several years before the time of his mother’s death and he survived without any additional care.

7 of the 11 orphans were adopted: one individual (4-year old female, no maternal siblings) was adopted by a non-kin parous adult female (see File S1 for definition of kin-relationships), but this only occurred after a prolonged period of 11-months during which the orphan was left on her own with no consistent care (for 2 or more days) from any group member, thus no temporary adoption took place in this time. In the other six cases, the orphans consisted of three sets of immature maternal siblings. In all cases, the older sibling (9–11 years) immediately provided care for the younger one (4–6 years), and we did not observe any allomaternal care from any mature group member, despite urgent needs and ample opportunities.

Survival amongst the seven adopted orphans (by unrelated adult or immature siblings) was significantly higher (100%) than that of the four non-adopted orphans (25%; Fisher’s exact test: p = 0.024).

We also re-examined the data reported at three other long-term research sites, Gombe, Mahale, and Taï [1]–[5], [20] (see Table 1) and compared results to Sonso. Across all four sites we found 34 cases in which allomaternal care was given to orphaned infants and two cases of temporary caregiving to infants whose mother disappeared but returned after a prolonged absence. In 20 of 36 cases (55.6%), adoption was by a non-kin adult individual, and in 2 of 36 cases (5.6%) by a related adult individual (1 father, 1 grandmother). In 14 of 36 cases (38.9%), adoption was by an older maternal sibling (both mature: N = 4; and immature-sibling adoption: N = 10).

**Table 1:** Individuals recorded as adopting (providing allomaternal-care to) orphans at long-term chimpanzee research sites, including cases of temporary care given during mother’s absence.

Chimpanzee community	Immature maternal sibling <12 yrs				Mature maternal sibling 12+yrs				Other kin				Non-kin			
	cases(n)	Adopterage (yrs)	time tocare (m)	Success(%)	cases(n)	Adopterage (yrs)	time tocare (m)	Success(%)	cases(n)	Adopterage (yrs)	time tocare (m)	Success(%)	cases(n)	Adopterage (yrs)	time tocare (m)	Success(%)
Sonso	3*	9–11	0–0	100	0	–	–	–	0	–	–	–	1	30	11	100
Gombe	5	8–10	0.25	60	2	17	0.25	100	1	>25	0	0	2	20–26	1	50
Mahale	–	–	–	–	–	–	–	–	–	–	–	–	4*	15+	0.25+	Unk
Taï	2	6–11	0.25	50	2	15–18	0.25–1	100	1	>15	0.25	0	13	13–35+	0.25–18	83
**Total**	**10**	**6–11**	**0–0.25**	**70**	**4**	**15–18**	**0.25–1**	**100**	**2**	**>15**	**0–0.25**	**0**	**20**	**13–35+**	**0.25–18**	**80**

The adopter’s age is given in years at time of the mother’s death or disappearance. Time to start of care is recorded in months (note cases recorded as ‘fast’ are listed as 1-week or less). Success rates are given as the percentage of orphans who lived for >1 year following their mother’s death. Taï: [1], [20]; Mahale: [3], [4]; Gombe: [2], [5].

* (n) includes one case of temporary adoption.

Across all four sites survival rates after one year were 80% for orphans adopted by non-kin adult adopters, 0% for orphans adopted by related adult adopters, 100% for orphans adopted by mature older sibling adopters and 70% for orphans adopted by immature sibling adopters. In the absence of the mother, maternal siblings (hereafter siblings, as there were no adoptions by paternal siblings), represent the only individuals with which an orphan is or has been permanently associated. Thus, they represent the individuals with whom the orphan has the closest social relationship. In a binary logistic regression considering the effect of orphan age, adopter age-class, and orphan-adopter social relationship (sibling vs. non-sibling) on 1-year survival, the model successfully classified 85% of cases (test of model versus intercept: N = 33 χ^2^ = 13.26, df = 3, p = 0.004; see Table 2). However, only orphan age predicted survival rate (p = 0.017): 100% of orphans aged 6-years or older and 95% of those aged 4-years or older survived; but only 42% of orphans under the age of 4-years, and 20% of orphans (one case, n = 5) under the age of 1-year survived. Neither adopter age (p = 0.827) nor orphan-adopter social relationship (p = 0.944) affected survival.

**Table 2:** Binary logistic regression predicting survival at 1-year following mother’s death.

Predictor	B	Wald χ^2^	P	Odds Ratio
Orphan age	0.92	5.69	0.017	2.51
Adopter age class	−0.09	0.01	0.944	0.92
Relationship	−0.47	0.05	0.827	0.62

Predictor variables for survival success included in the model were Orphan age (in years), Adoptor age-class (infant, juvenile, sub-adult, adult) and the adopter-orphan Relationship as siblings vs non-siblings (Hosmer and Lemeshow χ^2^ = 9.62, df = 8, p = 0.29). The model correctly classified 50% of those who died before 1-year, and 96% of those who survived, for an overall success rate of 84.8%.

We found a significant difference between sibling and non-sibling adopters in the amount of time between the mother’s death and adoption taking place. It took a non-sibling adopter significantly longer to adopt an orphaned infant compared to an older maternal sibling (of any age) (maternal siblings mean = 0.3±0.2 months; non-siblings mean = 5.2±6.6 months; unpaired t-test: t = 2.77 df = 26 p = 0.0103) despite the fact that maternal sibling adopters were significantly younger than other adopters (non-sibling adopters: n = 12, range 13–35 years, mean = 22.6±7.8 years; a further n = 7 identified sub-adult/adult but exact age unavailable; maternal-sibling adopters: n = 14, range 6–18 years, mean = 11.4±3.8 years; unpaired t-test: t = 4.76 df = 24 p<0.0001).

Given the effectiveness of adoption by both mature individuals and immature siblings, we analysed all cases of adoptions across the different field sites for the presence or absence of kin members in the group at the time of adoption. Importantly, we found that in none of the 16 cases in which an orphan had an older maternal sibling present was it adopted by an unrelated individual, even when the only older maternal sibling present was also an immature individual (which occurred in 11/16 cases). Our own data and the data of all long-term sites thus show that orphaned infant chimpanzees are first and foremost adopted by maternal siblings, regardless of age, if they are available. Where maternal siblings are not available, unrelated mature individuals may then adopt.

## Discussion

Our study shows that, across the major East African chimpanzee study sites, kin adoption takes priority, even if the kin-adopters are themselves still immature and mature non-kin adopters are available, suggesting that the ‘default’ pattern of adoption in chimpanzees is based on mutual support between orphaned siblings. Adoption by unrelated mature individuals does happen, and indeed was the most frequently observed type of adoption in the total data set and for the Tai community. Importantly, however, this type of adoption only occurs if no maternal siblings are available and often only after significant time periods (e.g. weeks or even months), despite ample availability of adopters and despite the orphan’s urgent need for care. One reason why the patterns we describe may have been overlooked in previous data is that the definition of adoption was limited to ‘mature’ individuals, 12 years or older [1]. Indeed, parenthood, while not impossible, very rarely occurs in immature wild chimpanzees (typical age of first offspring: females wild: 14–15 years [2], [3], [20]; captivity: 9–11 years [28]; males: >14 years [2], [20]. This fact renders it even more extraordinary that immature individuals not only adopted their younger siblings, but also were as successful as mature individuals when they did so. We suggest that rather than restricting the definition of adoption to ‘mature’ individuals [1], adoption by immature individuals should be included. We also suggest that, in future studies, a wider definition of adoptive care should be employed, including temporary adoption.

Our study suggests that it is the social (rather than biological) relationship that appears to play the key factor in an individual’s decision to adopt. Although there is some evidence that male chimpanzees may be able to recognise their own offspring, they show little or no preference to associate with them in the wild [18], [19]. A single case of paternal adoption was recorded in one Taï community [1]. However, given the large number of adoptions that occurred in the different Taï communities this may have been a chance event unrelated to paternal kin recognition. Similarly, adoption by paternal siblings has not yet been observed, although paternity data are often incomplete. Although both paternal and maternal siblings are similarly biologically related, they maintain very different social relationships. Paternal siblings typically behave like unrelated individuals [18], whereas maternal siblings typically form strong social bonds, especially when they are immature; during this time, they are permanently associated, and they travel with each other and sleep near each other every day until independent of their mother. Despite ample cases of sibling adoption we found no evidence for adoption by a paternal sibling (see Table S1), suggesting that the social-bonds that exist before the death of their mother play a key factor in the onset of adoptive behaviour. This hypothesis is further supported by the interesting exception to maternal sibling adoption in the Sonso community, where the orphan ‘Polina’ was not adopted by her maternal brother ‘Pascal’. The two siblings were separated by an unusually long interbirth interval of six years, and at the time of their mother’s death ‘Pascal’ had already been associating with the adult males of the community for several years, and only infrequently spent time with his mother and ‘Polina’.

We observed only one case of non-kin adoption in Sonso, although non-kin adoptions formed the majority of cases in the dataset. However, non-kin adoptions took significantly longer than kin adoptions to take place, and only mature non-kin individuals provided allomaternal care. A likely explanation is that the propensity to provide allomaternal care is related to the number of positive social interactions between the carer and the orphan, prior to the mother’s death, with the pre-existing social bonds between maternal siblings expediting this process. If no maternal-sibling adoption occurs, orphans are likely to interact with multiple community members [2], developing new social bonds through repeated positive interactions. These likely take time to form, hence the significantly longer period before adoption takes place if maternal siblings are not present.

In the Taï forest, the adoption of orphans by non-kin was particularly prevalent. Indeed, Taï is the only site (where both kin and non-kin adoption data are available) where the majority of adoptions were by non-kin (Sonso = 1 of 4; Gombe = 2 of 10; Tai = 13 of 18; see Table 1). Although group size was similar across communities (40–100 individuals: [1]–[3], [20], [29]–[31]), Taï chimpanzee communities are particularly cohesive with high rates of social interaction between most individuals [31]. Several factors affect chimpanzee group cohesion [32], [33]. The relatively high rates of regular social contact with non-kin individuals in Taï may result in interactions more similar to those of kin interactions in communities such as Sonso or Gombe, and may underpin the regular adoption of non-kin orphans by Taï individuals.

From our own and others’ observations it seems obvious that adopting an infant carries a significant cost for the adopter [1]–[4]. At the same time, it has been more difficult to confirm the benefit of adoption for the orphaned individuals. In the previous study in the Taï chimpanzees, adoption did not appear to increase likelihood of survival; however, this was likely due to the high rate of mortality, irrespective of the level of care experienced [1].

With this study, we are able to show that there is a significant increase in survival of adopted over non-adopted orphans, by adding data from communities with lower levels of mortality due to predation and disease. In the Sonso community, no orphaned infant or juvenile survived without the long-term care of another group member. In two cases the orphans disappeared with their mother and thus their deaths may have been linked, contrasting with the 100% survival of the adopted orphans. While adoption cannot guarantee survival, the potential benefits for the orphan are immense. Beyond increasing survival, adoption may also mitigate the loss of the important mother-infant bond, and allow the orphan an opportunity for ‘normal’ social development [2], [34]. Whether or not adoption in chimpanzees may be correctly classified as altruism remains unclear, given that adopters may derive a potential long term benefits from gaining a future ally [1]. However, female chimpanzees typically emigrate from their natal communities, suggesting that adopters should preferentially adopt male orphans if they want to gain a future ally [1]. In our data, 11 of the 20 orphans adopted by an un-related individual were females, suggesting that the ‘long-term social allies’ hypothesis carries little weight.

To conclude, chimpanzees display a range of prosocial behaviour towards orphaned individuals in their community, including adoption by relatives, adoption by non-relatives, and temporary adoption. In contrast to the results from West Africa [1] adoption by unrelated individuals was in the minority in East Africa. We suggest that the ‘default’ pattern is for orphans to associate with a maternal sibling, even if he or she is still immature, provided they are available. We further suggest that social bonds, rather than biological relatedness, underpin the adoption of orphaned chimpanzees, which are strongest between maternal siblings. If maternal siblings are unavailable, the orphans may be forced to develop social bonds with non-kin individuals, which may eventually lead to adoption. In socially cohesive groups, where non-kin individuals are more likely to have developed social bonds, adoption by non-kin individuals may thus be expedited.

In human populations, cases of parental behaviour between immature orphan siblings are usually referred to as ‘child-headed households’ [27], a common theme and leitmotif in fiction writing (e.g. ‘The Baudelaire children’). Our study shows that adoption by siblings, irrespective of age, is not only an important aspect of adoptive behaviour in humans [35], [36] but also in wild chimpanzees.

## Supporting Information

Table S1 **Immature orphans (<12 years) recorded in the Sonso community 1990–2013.** All recorded individuals in the Sonso chimpanzee community orphaned under 12-years of age. Details are provided of all known maternal and paternal kin in the community, together with the type and duration of any long-term care given.
(DOCX)

File S1 **Supplementary Results.** Detailed descriptions of the adoption and non-adoption of orphans in the Sonso community from 1990 to 2013.
(DOCX)
