# Supplementary material for: ‘Adoption’ by Maternal Siblings in Wild Chimpanzees
Source: PLoS One. 2014 Aug 1;9(8):e103777. doi: 10.1371/journal.pone.0103777 (PMC4118915; doi:10.1371/journal.pone.0103777)
Supplement: Table S1 — Immature orphans (<12 years) recorded in the Sonso community 1990–2013. All recorded individuals in the Sonso chimpanzee community orphaned under 12-years of age. Details are provided of all known maternal and paternal kin in the community, together with the type and duration of any long-term care given. (DOCX) [file pone.0103777.s001.docx]

**Table S1. Immature orphans (<12years) recorded in the Sonso community 1990-2013**

| **Orphan** | | | | | **Seen following mother’s death?** | **Known kin in community**  **(age at time of mother’s death)** | **Long-term care given (care-giver)?** | **Duration of care** |
| --- | --- | --- | --- | --- | --- | --- | --- | --- |
| **Name** | **Sex** | **Age y(m)** | **Year** | **Mother** |  |  |  |  |
| Biso | f | 4 | 1994 | Bwera | N | *Maternal: none known*  Paternal siblings and father unknown | No opportunity | No opportunity |
| Bob | m | 11 | 2001 | Ruda | Y | *Maternal sister (Rachel) 4yrs*  Paternal sister (Katia) 3yrs | N (adopts sibling) | 6yrs (until Bob’s death, Rachel then 11.5yrs at time, becomes independent). |
| Rachel | f | 4(4) | 2001 | Ruda | Y | *Maternal brother (Bob) 11yrs*  Father (Nkojo) 33yrs  Paternal brother (Zefa) 19yrs  Paternal brother (Gashom) 14yrs  Paternal brother (Simon) 9yrs | Y (Bob) |  |
| Clint | m | 4 | 2006 | Clea | N | Paternal sister (Bahati) 11yrs  Paternal sister (Nora) 10yrs  Paternal sister (Kana) 8yrs  Paternal sister (Ramula) 4yrs  Paternal brother (Zak) 4yrs  Paternal sister (Kuki) 3yrs | No opportunity | No opportunity |
| Zalu | m | 11 | 2007 | Zana | Y | *Maternal brother (Zed) 6yrs*  Paternal sister (Anna) 17yrs  Paternal brother (Kato) 14yrs  Paternal sister (Kewaya) 24yrs  Paternal brother (Pascal) 9yrs | N (adopts sibling) | Cares for younger brother  6yrs; both still in community, Zed independent as of 2013. |
| Zed | m | 6(3) | 2007 | Zana | Y | *Maternal brother (Zalu) 11yrs*  Father (Zefa) 25yrs  Paternal brother (James) 1yr  Paternal brother (Klauce) 1yr | Y (Zalu) |  |
| Polina | f | 4 | 2008 | Polly | Y | *Maternal brother (Pascal) 10yrs*  Paternal siblings and father unknown | N* | n.a |
| Pascal | m | 10 | 2008 | Polly | Y | *Maternal sister (Polina) 4yrs*  Paternal sister (Kewaya) 25yrs  Paternal sister (Anna) 18yrs  Paternal brother (Kato) 15yrs  Paternal brother (Zalu) 13yrs | N* | n.a. |
| Sharlot | f | 3 | 2010 | Sabrina | Y | *Maternal: none known*  Father (Musa) 19yrs  Paternal sister (Goria) 4yrs | Y (Wilma, non-kin) | 1yr (ongoing) |
| Ramula | F | 9 | 2012 | Ruhara | Y** | *Maternal brother (Nick) 30yrs*  *Maternal sister (Rose) 15yrs*  *Maternal sister (Rafia) 5yrs*  Paternal sister (Kana) 14yrs  Paternal sister (Kuki) 9yrs | N (becomes carer)  Assisted by older siblings (Nick, Rose) and adult maternal ally (Melissa) | 1^st^ occasion 63 days (maternal siblings Ramula, Rose, Nick all gave care; non-kin parous adult female Melissa allows them to travel with her at times)  2^nd^ occasion (2-3weeks) Ramula was main carer for younger sister Rafia (sisters travel at times with maternal brother Nick and non-kin Melissa). |
| Rafia | F | 5 | 2012 | Ruhara | Y** | *Maternal brother (Nick) 30yrs*  *Maternal sister (Rose) 15yrs*  *Maternal sister (Ramula) 9yrs*  Father (Zefa) 30yrs  Paternal brother (Zed) 11yrs  Paternal brother (James) 6yrs  Paternal brother (Klauce) 6yrs  Paternal brother (Kaspa) 4yrs | Y (Ramula)  Assisted at times by older siblings (Nick, Rose) and adult maternal ally (Melissa) |  |

* Polly was a peripheral female who was only rarely seen with the main group, Pascal was regularly seen to move with the core group, without his mother, from approximately the time his sister Polina was born; ** Ruhara was temporarily absent for several months while on extended consortship. An extended absence from the core group, while the dependent children remain behind had never been recorded in the Sonso community, and she was presumed to be dead at the time.
