# Supplementary material for: ‘Adoption’ by Maternal Siblings in Wild Chimpanzees
Source: PLoS One. 2014 Aug 1;9(8):e103777. doi: 10.1371/journal.pone.0103777 (PMC4118915; doi:10.1371/journal.pone.0103777)
Supplement: File S1 — Supplementary Results. Detailed descriptions of the adoption and non-adoption of orphans in the Sonso community from 1990 to 2013. (DOCX) [file pone.0103777.s002.docx]

**File S1. Supplementary Results**

Paternity

Paternity was confirmed for 7 of the 11 Sonso orphans (table S1). In one case, paternity was unknown as the orphan was not genotyped (Biso), but no adoption took place.

Non-adoption

Three of the six orphans were not adopted following their mother’s death. In two cases the infants disappeared with the mother with no apparent opportunity for adoption. In one of these two cases paternal siblings were present in the community, including two independent individuals (Clint; see table S1). In the other case, no known maternal or paternal kin were present at the time (Biso; table S1), although genetic data were not available at the time. The third orphan, Polina, was seen immediately after her mother’s death. In addition, an infant that resembled Polina was seen on several occasions over the subsequent 2-year period before disappearing completely. During this time, the infant was seen moving with different females and their families but was not adopted. Although Polina had an older maternal brother in the community (Pascal, 10-years; independent at approximately the time of Polina’s birth) he did not adopt her, nor was he observed to engage in any parental behaviour towards Polina when she was occasionally seen travelling with the core group.

Adoption of kin

Three of the six orphans were adopted, two of them by siblings. In both cases the adopters were older maternal siblings who had only recently reached subadulthood (both age 11yrs) and still moved regularly with their mother and younger sibling, before their mothers’ death.

*Rachel by brother Bob:* Following the death of their mother Ruda (†29.10.2001, severe chronic peritonitis), 4y 4m old infant female Rachel (*June 1997) was adopted by her 11 year old, still dependent brother Bob (*1990). Rachel was last seen May 2011 aged 14yrs; she is assumed to have emigrated to a new community.

*Zed by brother Zalu:* Following the death of their mother Zana (†03.08.2007, intra-community attack), 6y 3m old infant male Zed (*May 2001) was adopted at by his 12 year old, still dependent brother Zalu (*June 1995). Both Zed and Zalu had become independent males in the Sonso community at the time of this study.

Adoption of non-kin

*Sharlot by non-kin female Wilma:* Following the death of her mother Sabrina († 28/29.08.2011), 4y 0m old infant female Sharlot was found alone in a tree adjacent to her mother’s body. Members of the Sonso community joined her on the 30.08.2011 and she moved off together with them. During the subsequent 11-month period, from September 2010 to June 2011, Sharlot was regularly observed to travel, rest, play and feed with core members of the Sonso community, frequently moving with adult and sub-adult males. During this time non-kin male community members were observed to defend her from aggression (Nick α-male, Zefa γ-male), carry her ventrally (Nick α-male), interact with her affiliatively when distressed (Zefa γ-male, Zed), groom her (Simon), embrace her (Zalu, Zefa γ-male), and share food with her (Musa β-male and her father, Kato). From 5th June 2011 Sharlot was observed moving with Wilma, a primiparous female whose only offspring died 10 years previously at the age of 5. At the time of the study, they had been moving together for over 18 months. We observed regular maternal behaviour in Wilma (carrying, defending, grooming, nesting) and offspring behaviour in Sharlot (requesting carrying, attempted suckling, grooming, nesting). We have been unable to confirm if the attempts to suckle are successful, and it is not clear that Wilma, 16-years after last giving birth, would be able to produce breast milk.

Temporary care of non-orphans

*Ramula and Rafia by sister Rose:* Following two temporary disappearances of their mother Ruhara (21th Jun -7^th^ Aug 2012; 23^rd^ Aug - Sept 2012 (exact date of return not recorded), both times on consortship with gamma-male Zefa), 5y 0m infant female Rafia (*July 2007) and 9y 0m juvenile sister Ramula (born Sept 2002), both still dependent, were seen to move with their 14y old independent maternal sister Rose (*1997), their 30y old independent maternal brother Nick (alpha-male, *1982), and the family of the multi-parous female Melissa (no confirmed kin relationship).

On Ruhara’s first return to the community the three daughters continued to move independently, apart from her, for almost two weeks before joining her on the morning of the day that she was taken again on a second consortship by Zefa (23.08.2012), disappearing with her daughters. 11 days later, Ramula and Rafia returned alone to the community, more than a week before their older sister Rose and mother Ruhara returned. During this period, the two younger sisters again moved with several individuals, including their older brother Nick and the adult female Melissa.
